# Machine Learning-Based Models Enhance the Prediction of Prostate Cancer

**DOI:** 10.3389/fonc.2022.941349

**Published:** 2022-07-06

**Authors:** Sunmeng Chen, Tengteng Jian, Changliang Chi, Yi Liang, Xiao Liang, Ying Yu, Fengming Jiang, Ji Lu

**Affiliations:** ^1^ Department of Urology, The First Hospital of Jilin University, Changchun, China; ^2^ School of Business and Management, Jilin University, Changchun, China

**Keywords:** prostate cancer, machine learning, prostate-specific antigen, prostate biopsy, prediction models

## Abstract

**Purpose:**

PSA is currently the most commonly used screening indicator for prostate cancer. However, it has limited specificity for the diagnosis of prostate cancer. We aim to construct machine learning-based models and enhance the prediction of prostate cancer.

**Methods:**

The data of 551 patients who underwent prostate biopsy were retrospectively retrieved and divided into training and test datasets in a 3:1 ratio. We constructed five PCa prediction models with four supervised machine learning algorithms, including tPSA univariate logistic regression (LR), multivariate LR, decision tree (DT), random forest (RF), and support vector machine (SVM). The five prediction models were compared based on model performance metrics, such as the area under the receiver operating characteristic curve (AUC), accuracy, sensitivity, specificity, calibration curve, and clinical decision curve analysis (DCA).

**Results:**

All five models had good calibration in the training dataset. In the training dataset, the RF, DT, and multivariate LR models showed better discrimination, with AUCs of 1.0, 0.922 and 0.91, respectively, than the tPSA univariate LR and SVM models. In the test dataset, the multivariate LR model exhibited the best discrimination (AUC=0.918). The multivariate LR model and SVM model had better extrapolation and generalizability, with little change in performance between the training and test datasets. Compared with the DCA curves of the tPSA LR model, the other four models exhibited better net clinical benefits.

**Conclusion:**

The results of the current retrospective study suggest that machine learning techniques can predict prostate cancer with significantly better AUC, accuracy, and net clinical benefits.

## Introduction

Prostate cancer (PCa) is the second leading malignancy in men and the fifth leading cause of cancer mortality in men worldwide (1). Although PSA is still the most commonly used screening tool for prostate cancer, it has been controversial in recent decades (2). It is suggested that PSA screening improves the detection rate of localized and less aggressive prostate cancers but also reduces the proportions of advanced PCa and PCa-specific mortality (3–5). However, due to the obvious overlap of PSA levels in various conditions, such as benign prostatic hyperplasia, prostatitis, and prostate cancer, the specificity of PSA screening is low, which leads to a plethora of unrelated diseases for prostate biopsy (2). These unnecessary prostate biopsies result in not only a significant waste of medical resources but also an increased incidence of sepsis, which can be life-threatening to patients (6). Therefore, there is a need for a new convenient method to improve the diagnostic ability of PCa.

Machine learning is a branch of artificial intelligence (AI) in which machines are programmed to learn patterns from data, and the learning itself is based on a set of mathematical rules and statistical assumptions. It is widely used in biology because of its enormous advantages in dealing with large datasets (7, 8). It has also been rapidly developed and applied in the medical field, especially in the construction of predictive models (9). Therefore, using machine learning to construct PCa prediction models would be a feasible and promising approach.

In this study, we constructed generalizable machine learning predictive models to improve the accuracy of PCa risk assessment by using objective parameters present in electronic medical records and then evaluated their performance.

## Methods

### Data Sources

A total of 789 male patients in the First Hospital of Jilin University who underwent transrectal ultrasound-guided prostate biopsy from January 2013 to January 2021 were included. Indications for prostate biopsy included serum tPSA >4 ng/ml, abnormal digital rectal exam (DRE), or imaging findings suggestive of suspected prostate cancer. All patients underwent systematic biopsy with 10-12 cores. Patients with one of the following criteria were excluded from the study: taking medications that could affect serum PSA levels, unclear results of the prostate biopsy, and significant abnormal values or missing data. A final total of 551 patients were included in the study. All patient data were collected through electronic medical records, including age, BMI, hypertension, diabetes, total PSA (tPSA), free PSA (fPSA), the ratio of serum fPSA to tPSA (f/tPSA), prostate volume (PV), PSA density (PSAD), neutrophil-to-lymphocyte ratio (NLR), and pathology reports of prostate biopsy. All examinations were completed within one week before prostate biopsy. PSAD is the ratio of tPSA to PV. The calculation of PV was calculated by the following formula: maximal transverse diameter × maximal anterior-posterior diameter × maximal superior-inferior diameter × 0.52.

### Model Development

R software (version 4.1.4, https://www.rproject.org/) was used to develop machine learning models. A total of five prediction models were constructed by dividing the data into a training dataset and a test dataset at a ratio of 3:1. The pathologic type of PCa or other benign disease was used as the dichotomous variable, and other variables were all used as continuous variables. For logistic regression (LR) model construction, the best predictive variables were first screened in the training dataset using stepwise regression, association plots between the variables were made to understand the magnitude of the association between the variables, and the presence or absence of collinearity between variables was judged according to the variance inflation factor (VIF). Then, LR models were constructed using the “lrm” function in the “rms” package. For the decision tree (DT) model, we used the “rpart” package for training, using two hyperparameters, the complexity parameters cp and spilt. The initial cp value was set to 0.001, and then the best cp value was found and pruned based on the best cp value. The input variables were obtained through the selection of important features, and the best DT model was then output. The random forest (RF) model screened the optimal input variables by significant feature selection. The RF model was trained using the “randomForest” package in R software, using two hyperparameters, ntree and mtry, which were set at 500 and 6, respectively. The support vector machine (SVM) model was filtered by the “caret” package for important features. Training was performed using the “e1071” package, using a Gaussian kernel function and setting the two hyperparameters, cost and gamma, to 1 and 0.1, respectively.

### Model Performance Evaluation

The performance of the developed models was validated using a test dataset in a process that was completely independent of the algorithm training. The performance of the five models was then evaluated by comparing four metrics: the receiver operating characteristic (ROC) curve and its corresponding area under the curve (AUC) and the accuracy, sensitivity, and specificity. The calibration curve was used to evaluate the calibration of the model, the Brier score was used to assess the calibration, the Hosmer–Lemeshow goodness-of-fit test was used to judge whether there was a significant difference between the observed and predicted values, and clinical decision curve analysis (DCA) was used to assess the net benefit of the model.

### Data Analysis

For comparative analysis between two samples, Student’s t test was used for normally distributed continuous variables, and the Mann–Whitney U test was used for categorical variables with nonnormal continuous variables. Continuous variables in the data were expressed as medians and IQRs or means and SDs, categorical variables were expressed as frequencies and percentages, and the bilateral significance level for the left-right test was set at 5% (p<0.05).

## Results

### Baseline Patient Characteristics

**Table 1** shows the baseline characteristics of the patients. A total of 302 (54.8%) of the 551 patients were diagnosed with PCa. The PCa detection rate in patients ≥65 years old was higher than patients <65. The mean levels of tPSA, fPSA, and PSAD were significantly higher in the PCa group than in the non-PCa group. When tPSA>4 ng/ml, the PCa detection rate increased with increasing tPSA. In the subgroups of 4<tPSA<10, 10≤tPSA<20, 20≤tPSA<100, and tPSA≥100, the detection rates were 18.6%, 26.2%, 54.2%, and 97.2%, respectively. The mean neutrophil count, PV, and BMI were lower in the PCa group than in the non-PCa group. No significant differences in other variables were found between two groups.

**Table 1:** Characteristics of Patients, stratified by biopsy outcomes.

	Total (n=551)	Non-PCa (n=249)	PCa (n=302)	PCa detection (%)	p value
**Age,year(n,%)**					< 0.001
**<65**	157 (28.5)	96 (38.6)	61 (20.2)	38.9	
**≥65**	394 (71.5)	153 (61.4)	241 (79.8)	61.2	
**BMI,kg/m2(SD)**	23.6 ± 3.2	24.0 ± 3.0	23.3 ± 3.4		0.013
**Hypertension,(n,%)**					0.059
**No**	416 (75.5)	178 (71.5)	238 (78.8)	57.2	
**Yes**	135 (24.5)	71 (28.5)	64 (21.2)	47.4	
**Diabetes,(n,%)**					0.603
**No**	511 (92.7)	233 (93.6)	278 (92.1)	54.4	
**Yes**	40 (7.3)	16 (6.4)	24 (7.9)	60.0	
**Neutrophil count, 10^9^/L(IQR)**	3.7 (2.9-4.8)	4.0 (3.1-5.0)	3.7 (2.8-4.6)		0.024
**Lymphocyte count, 10^9^/L(SD)**	1.8 ± 0.7	1.8 ± 0.6	1.8 ± 0.7		0.798
**NLR,(IQR)**	2.1 (1.5-3.0)	2.1 (1.5-3.3)	2.0 (1.5-2.9)		0.150
**Median tPSA, ng/ml(IQR)**	32.0 (14.5-100.0)	16.2 (10.6-26.1)	88.8 (34.0-123.2)		< 0.001
**tPSA, ng/ml,(n,%)**					< 0.001
**≤4**	13 (2.4)	10 (4)	3 (1)	23.1	
**4<tPSA<10**	59 (10.7)	48 (19.3)	11 (3.6)	18.6	
**10≤tPSA<20** **20≤tPSA<100**	122 (22.1) 212 (38.5)	90 (36.1) 97 (39)	32 (10.6) 115 (38.1)	26.2 54.2	
**≥100**	145 (26.3)	4 (1.6)	141 (46.7)	97.2	
**fPSA,ng/ml(SD)**	13.6 ± 31.3	3.3 ± 5.8	22.2 ± 39.9		< 0.001
**f/tPSA,(n,%)**					0.280
**<0.16**	390 (70.8)	170 (68.3)	220 (72.8)	56.4	
**≥0.16**	161 (29.2)	79 (31.7)	82 (27.2)	50.9	
**PV,cm^3^(IQR)**	55.3 (37.2-79.0)	65.2 (44.7-91.5)	48.9 (34.3-70.3)		< 0.001
**PSAD,ng/ml/cm^3^(IQR)**	0.6 (0.3-1.6)	0.3 (0.2-0.4)	1.5 (0.7-2.7)		< 0.001

PV, prostate volume; PSAD, prostate-specific antigen density; BMI, body mass index; NLR, neutrophil-to-lymphocyte ratio; tPSA, total prostate specific antigen; fPSA, free prostate specific antigen; PCa, prostate cancer; Non-PCa, non-prostate cancer.

### LR Algorithm-Based PCa Prediction Model

First, the tPSA univariate LR model was constructed in the training dataset by including only one single factor, tPSA. This model showed that tPSA was positively correlated with the diagnosis of PCa (coefficient=0.034). Then, a multivariate LR model was constructed by including all variables through a stepwise regression method. When the VIC value reached the minimum value, a total of seven best predictive variables were selected, including age, tPSA, fPSA, PV, NLR, peripheral blood neutrophil count and lymphocyte count. The two LR models are shown in **Supplementary Table 1**. In the multivariate LR model, age, tPSA, and fPSA were positively correlated with PCa, while PV and neutrophil count were negatively correlated with PCa (**Supplementary Figure 1**). In addition, there was a significant association between peripheral blood neutrophil count and NLR, tPSA and fPSA, suggesting their respective possible collinearity. The variance inflation factor (VIF) was subsequently calculated for verification, and all VIF values were less than 5, indicating no collinearity between any of the variables.

### DT Algorithm-Based PCa Prediction Model

The optimal cp value for the DT model was 0.008. Based on the corresponding ranking of important features, the final input variables for the DT model were age, tPSA, fPSA, PV, PSAD, NLR, f/tPSA, and biopsy results (**Supplementary Figure 2A**). The process and results of model classification are shown in **Supplementary Figure 2B**. This model correctly classified 87.7% (363/414) of the cases in the training dataset.

### RF Algorithm-Based PCa Prediction Model

After ranking the important features of the RF model, seven features with the highest predictive accuracy were selected as the input features, including age, tPSA, fPSA, PV, PSAD, NLR, and peripheral blood neutrophil count (**Supplementary Figure 3A**). The error of the model gradually decreased as the number of decision trees increased, and the minimum error value of the RF model was reached when the number of decision trees was 313 (**Supplementary Figure 3B**).

### SVM Algorithm-Based PCa Prediction Model

The best SVM model was screened by the “rfe” function in the caret package using a 10-fold cross-validation method. The number of variables was screened one by one from 1 to 12, and the best model was obtained when the number of variables was 5. The input variables at this time were age, PSAD, tPSA, fPSA, and PV.

### Performance of the Developed Models

**Figure 1:**
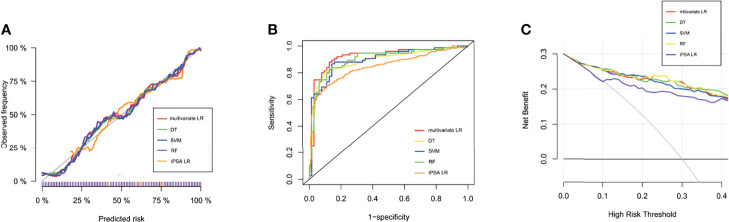
Performance of machine learning models. **(A)** Calibration curves of five prediction models in the training dataset. The predicted probabilities are plotted on the X-axis, and the actual probabilities are plotted on the Y-axis. **(B)** The ROC curves of the tPSA LR, multivariate LR, DT, RF, and SVM models in the test dataset. **(C)** Clinical decision curve analysis (DCA) of the five models in the test dataset.

In the training dataset, the RF and DT models performed particularly well in differentiation with the AUCs of 1.0 and 0.922, respectively (**Table 2**). The model with the lowest AUC was the tPSA univariate LR model (0.842). The calibration in all five models was very good, which suggested that the predicted values of the models were in high agreement with the actual values (**Figure 1A**). The Brier scores of the multivariate LR, DT, RF, SVM and tPSA LR models were 0.119, 0.122, 0.121, 0.118 and 0.154, respectively. The p values for the Hosmer–Lemeshow test were all greater than 0.05, indicating that there was no statistical bias in the near-perfect fit between the predicted and actual values. Therefore, the models we constructed were valid and reliable.

**Table 2:** Diagnostic performance of different machine learning models.

Outcome	Dataset	tPSAlogisticregression	Multivariate logisticregression	Decision Tree	Random Forest	SupportVectorMachine
AUC	Training	0.842	0.910	0.922	1.00	0.884
	Test	0.846	0.918	0.886	0.898	0.895
Sensitivity(%)	Training	69.2	70.5	91.2	100	86.8
	Test	63.9	88.0	86.7	84.0	86.7
Specificity(%)	Training	88.8	95.2	83.4	100	70.6
	Test	93.2	87.1	69.4	79.0	85.5
Accuracy(%)	Training	78.0	81.6	87.7	100	79.5
	Test	77.1	87.6	78.8	81.8	86.1

All characteristics of samples were comparable in the training and test datasets (**Supplementary Table 2**). In the test dataset, the specificity of the tPSA LR model was highest, reaching 93.2%; however, the sensitivity and accuracy were relatively low, at 63.9% and 77.1%, respectively (**Table 2**). The sensitivity and accuracy of the multivariate LR model were improved significantly in the test dataset compared with the training dataset, although there was a slight decrease in specificity. The diagnostic performance of the SVM model was not outstanding in either the training or the test dataset, but most outcomes were improved in the test dataset. Thus, the multivariate LR model exhibited the best discrimination, and the extrapolation and generalization abilities of the multivariate LR and SVM models were relatively strong. In contrast, although the DT and RF models performed well in the training dataset, their performances in the test dataset decreased significantly. The corresponding ROC curves for the five models are shown in **Figure 1B**. To further evaluate the potential clinical benefits of these prediction models, we performed DCA curves using the test dataset (**Figure 1C**). All models demonstrated significant net benefits. Compared with the tPSA LR model, the other four models showed significantly higher net clinical benefits.

To evaluate the performance of constructed models in the subgroups of PSA of 4-10 ng/ml and 4-20 ng/ml, we used these two subgroups as the test datasets, and evaluated their AUCs, sensitivities, specificities, and accuracies (**Supplementary Tables 3, 4**). The diagnostic performances of the RF algorithm-based machine learning prediction model showed best in both subgroups. The AUCs were 0.856 and 0.94 respectively. Although the sensitivity decreased compared with it in the training dataset, the specificity and accuracy were still relatively high. The performance of other algorithms-based developed models were not outstanding.

## Discussion

In the past decade, PSA has been widely used as the most valuable diagnostic and prognostic marker for PCa (10). However, some studies have shown that less than 30% of men with PSA levels in the gray zone (4-10 ng/ml) have pathologically confirmed PCa, indicating that a large proportion of patients have undergone unnecessary biopsies and been overdiagnosed (11). In our study, the detection rate of PCa with tPSA in the gray zone was only 18.6%. Similar detection rates were reported in other studies (12–15). Even if PSA is not in the gray zone, for example, between 10 and 20 ng/ml, the detection rate in our study was only 26.2%. In recent years, DRE, PSAD, PSAV, 4Kscore, f/tPSA ratio, prostate health index (PHI) and age-specific PSA have been proposed as predictors for PCa (3, 16–18). However, it may be difficult to achieve good predictive results with any single factor.

In this study, we constructed prediction models of PCa based on machine learning algorithms. Four algorithms were used, and a total of five models were constructed. First, we constructed a univariate logistic regression model using tPSA. As shown in **Table 2**, in the test dataset, although the specificity of the tPSA LR model reached 0.932, its sensitivity was decreased to only 0.639. The AUC of this model was only 0.846, which was significantly lower than that of the other models, showing the limitations of using tPSA alone as a predictor of PCa. Among them, the multivariate LR model had higher specificity, sensitivity, accuracy, and AUC in the test dataset, showing good predictive ability. Therefore, the shortcomings of low sensitivity and accuracy of the tPSA LR model were complemented very well by the inclusion of more variables. In our study, the multivariate LR model also had outstanding extrapolation and generalization ability due to the small number of changes between the training and test datasets. As a model similar to traditional statistical analysis methods, the results of the LR model had strong interpretability, which could help clinicians predict PCa based on relevant factors. The output of the DT model was similar to the clinical pathway. It is a clinician- and patient-friendly model and has strong clinical operability. Anyone can follow the predicted model from the root node to the leaf node to make decisions. However, in our study, the performance of the constructed DT model decreased significantly when it was validated in the test dataset and had very low specificity. The RF and SVM models had average diagnostic performance in our study, and their “black box” style reduced the clinical interpretability slightly.

The PCa detection rate in patients with PSA<20 ng/ml was relatively low in our study. Thus it is important to predict PCa in this population. The RF model performed best in both subgroups PSA of 4-10 ng/ml and 4-20 ng/ml. This suggested that the machine learning models we constructed based on overall population might also be applicable in patients with PSA ranged 4-10 or 4-20 ng/ml. In the training dataset, the RF model outperformed other models with the AUC of 1.0, while its performance decreased significantly in the test dataset. But in these two PSA subgroups, about 72% of samples overlapped with the samples in the training dataset, and were involved in the construction of models. That may lead to the outperformance of RF model rather than other models in PSA subgroups. Future study should focus on this population and develop more accurate machine learning models.

In recent years, some studies on the prediction of PCa by machine learning models have been published. In the study of Peter Ka-Fung Chiu et al., four variables, PSA, DRE, PV and transrectal ultrasound findings, were included, and SVM, LR, and RF models were constructed. All models were shown to have better prediction for PCa and clinically significant PCa than PSA and PSAD alone (19). Similarly, Nitta et al. suggested that compared to the AUCs of the PSA level, PSAD, and PSAV alone, the AUCs of artificial neural network (ANN), RF, SVM machine learning models were all improved when age, PSA level, PV, and white blood cell count in urinalysis were incorporated (20).In a study including patients with tPSA<10 ng/ml, a PSA-based machine learning model was constructed based on dense neural network with an AUC of 0.72, which was improved compared to PSA alone, age, fPSA and f/tPSA alone (21). In another study, multiparametric MRI (mpMRI) combined with other characteristics of patients was included to construct a machine learning model. The SVM and RF yielded similar diagnostic accuracy and net benefit and spared more biopsies at 95% sensitivity for the detection of clinically significant PCa compared with logistic regression (15).

Recently, a meta-analysis showed that the performance of selectMDx test in urine was comparable to that of mpMRI with regards to PCa detection. The AUC of selectMDx only was 0.854, and the AUC of one or both positive finding with selectMDx and/or mpMRI could reach 0.909. However, the multivariate LR model in our study still shows strength with AUC of 0.918 in test dataset (22). The integrative machine learning model was constructed to predict negative prostate biopsy utilizing both radiomics and clinical features. Although that model got high performance with negative predictive value of 98.3%, the AUC, sensitivity, and specificity were 0.798, 83.3%, and 75.2%, respectively, which were relatively lower compared with those in our models (23). Considering that all variables in our models are objective indicators, reducing possible errors of manual evaluation, we believe that the advantages of our models are more obvious.

Although machine learning-based models for PCa prediction have been constructed and validated, there are several limitations in our study. First, our study is retrospective in nature and may be potentially biased, and the sample size may not be adequate for some machine learning algorithms. Second, both the training and test datasets were from the same hospital, so further external validation at other centers is needed to confirm the findings. Third, some important factors or variables were not included in our models. For example, it has been shown that mpMRI provides more imaging information than conventional ultrasound, not only improving the detection of PCa but also helping to distinguish clinically significant PCa (24). Limited by insufficient data, mpMRI and its PI-RADS data were not included in our study. It is hoped that incorporating mpMRI into machine learning models may help to further improve the diagnostic performance of models in the future.

## Conclusion

In conclusion, by retrieving electronic medical records, we developed, validated, and compared machine learning models to predict PCa in the biopsy population. All models showed clinical benefits based on DCA. Multivariate LR, DT, RF, and SVM models were better than tPSA univariate LR. Among these models, multivariate LR performed best, with an AUC of 0.918 in the test dataset. Constructing machine learning-based models and predicting PCa is feasible. This could enhance the detection of PCa and help to avoid unnecessary prostate biopsy.

## Data Availability Statement

The raw data supporting the conclusions of this article will be made available by the authors, without undue reservation.

## Author Contributions

SC: Data analysis, Manuscript writing/editing. TJ: Data collection. CC: Data collection. YL: Data analysis. XL: Data analysis. YY: Data collection. FJ: Project development, supervision. JL: Project development, supervision, manuscript writing/editing. All authors contributed to the article and approved the submitted version.

## Funding

This study was funded by Science and Technology Development Project of Jilin Province, China (20200201315JC).

## Conflict of Interest

The authors declare that the research was conducted in the absence of any commercial or financial relationships that could be construed as a potential conflict of interest.

## Publisher’s Note

All claims expressed in this article are solely those of the authors and do not necessarily represent those of their affiliated organizations, or those of the publisher, the editors and the reviewers. Any product that may be evaluated in this article, or claim that may be made by its manufacturer, is not guaranteed or endorsed by the publisher.
