# Supplementary material for: Machine Learning-Based Models Enhance the Prediction of Prostate Cancer
Source: Front Oncol. 2022 Jul 6;12:941349. doi: 10.3389/fonc.2022.941349 (PMC9299367; doi:10.3389/fonc.2022.941349)
Supplement: Supplementary file 1 [file DataSheet_1.pdf]

Supplementary Table 1　Logistic regression analysis results on the training dataset

	Regression coefficient	Std.Error	P value	OR(95%CI)
**tPSA LR model**				
tPSA	0.034	0.004	<0.001	1.035(1.027,1.044)
constant term	-1.400	0.189	<0.001	0.247(0.169,0.354)
**Multivariate LR model**				
Age	0.077	0.017	<0.001	1.080(1.045,1.119)
tPSA	0.031	0.006	<0.001	1.032(1.020,1.045)
fPSA	0.053	0.027	0.049	1.055(1.006,1.121)
neutrophil.count	-0.311	0.109	0.004	0.733(0.563,0.890)
lymphocyte.count	0.427	0.265	0.107	1.533(0.927,2.721)
NLR	0.174	0.110	0.114	1.190(0.995,1.644)
prostate.volume	-0.031	0.005	<0.001	0.970(0.959,0.979)
constant term	-4.867	1.325	<0.001	0.008(0.001,0.097)

Supplementary Table 2 Characteristics of the Training and Test Datasets

	Total (n = 551)	Training (n = 414)	Test (n = 137)	P value
**Age,year(n,%)**				0.694
**<65**	157 (28.5)	123 (29.7)	34 (24.8)	
**≥65**	394 (71.5)	291 (70.3)	103 (75.2)	
**BMI,kg/m2(SD)**	23.6 ± 3.2	23.6± 3.2	23.5 ± 3.4	0.683
**Hypertension,(n,%)**				0.089
**No**	416 (75.5)	320 (77.3)	96 (70.1)	
**Yes**	135 (24.5)	94 (22.7)	41 (29.9)	
**Diabetes,(n,%)**				0.720
**No**	511 (92.7)	383 (92.5)	128(93.4)	
**Yes**	40 (7.3)	31 (7.5)	9 (6.6)	
**Neutrophil count,10^9/L(IQR)**	3.7 (2.9-4.8)	3.9 (3.1-4.9)	3.6 (2.8-4.4)	0.062
**Lymphocyte count,10^9/L(SD)**	1.8 ± 0.7	1.8 ± 0.6	1.8 ± 0.8	0.958
**NLR,(IQR)**	2.1 (1.5-3.0)	2.1 (1.6-3.1)	1.9 (1.4-2.8)	0.154
**Median tPSA,ng/ml(IQR)**	32.0 (14.5-100.0)	34.3 (15.3-100.0)	26.1 (12.8-100.0)	0.096
**tPSA,ng/ml,(n,%)**				
**≤4**	13 (2.4)	9 (2.2)	4 (2.9)	
**4<tPSA<10**	59 (10.7)	42 (10.1)	17 (12.4)	
**10≤tPSA<20**	122 (22.1)	90(21.7)	32 (23.4)	
**≥20**	357 (64.8)	271 (65.9)	86 (61.3)	
**fPSA,ng/ml(SD)**	13.6 ± 31.3	12.3 ± 24.1	17.8 ± 46.6	0.072
**f/tPSA,(n,%)**				0.531
**<0.16**	390 (70.8)	290 (70.0)	100 (73.0)	
**≥0.16**	161 (29.2)	124 (30.0)	37 (27.0)	
**PV,cm^3(IQR)**	55.3 (37.2-79.0)	56.5 (37.7-80.3)	49.8 (36.0-76.2)	0.335
**PSAD,ng/ml/cm^3(IQR)**	0.6 (0.3-1.6)	0.6 (0.3-1.6)	0.6 (0.2-1.6)	0.062
**PCa(n,%)**	302(54.8)	228(55.1)	74(54)	0.830

Supplementary Table 3 Performance of Constructed Models in Patients with PSA 4-10 ng/ml (n=59)

Machine Learning Models	AUC	Sensitivity (%)	Specificity (%)	Accuracy (%)
**tPSA LR**	0.54	36	81	73
**Multivariate LR**	0.68	82	69	71
**Decision Tree**	0.78	64	91	86
**Random Forest**	0.856	82	98	95
**Support Vector Machine**	0.73	82	65	68

Supplementary Table 4 Performance of Constructed Models in Patients with PSA 4-20 ng/ml (n=181)

Machine Learning Models	AUC	Sensitivity (%)	Specificity (%)	Accuracy (%)
**tPSA LR**	0.53	84	28	41
**Multivariate LR**	0.8	88	65	71
**Decision Tree**	0.83	65	86	81
**Random Forest**	0.94	79	96	92
**Support Vector Machine**	0.81	88	64	70

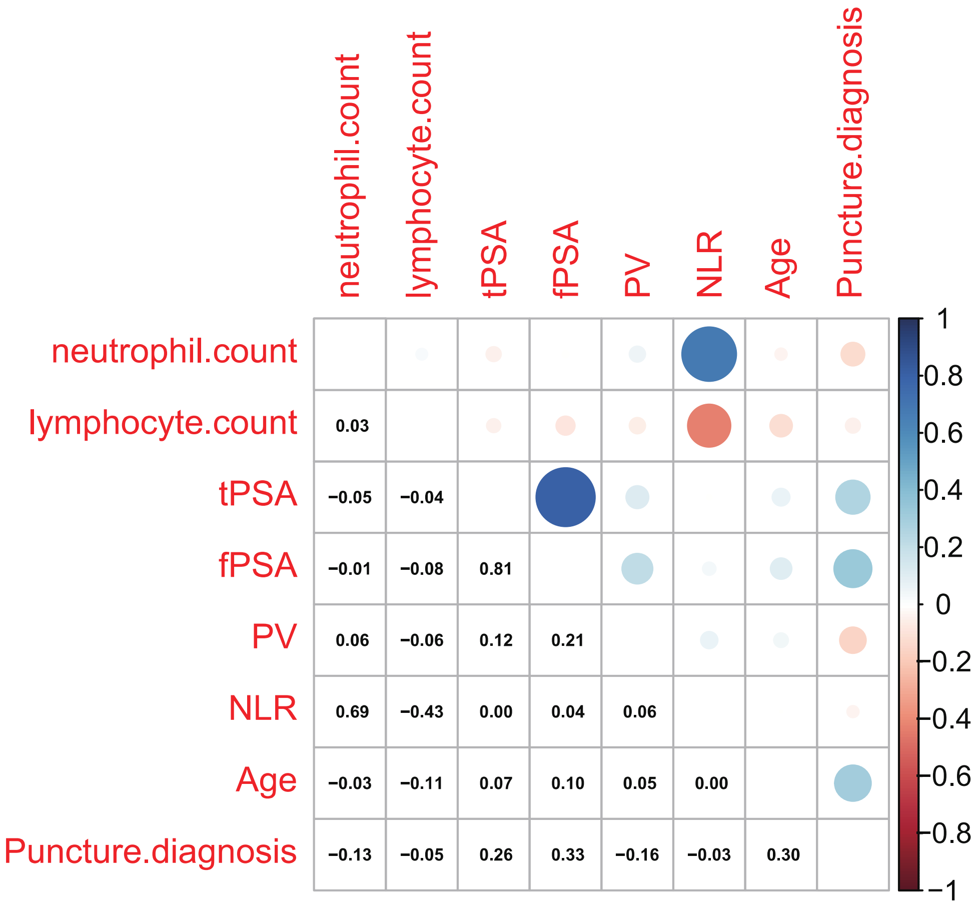

Supplementary Fig. 1 Association diagram between variables. The size and color of the circle indicate the strength of the association; blue is positive, and red is negative.

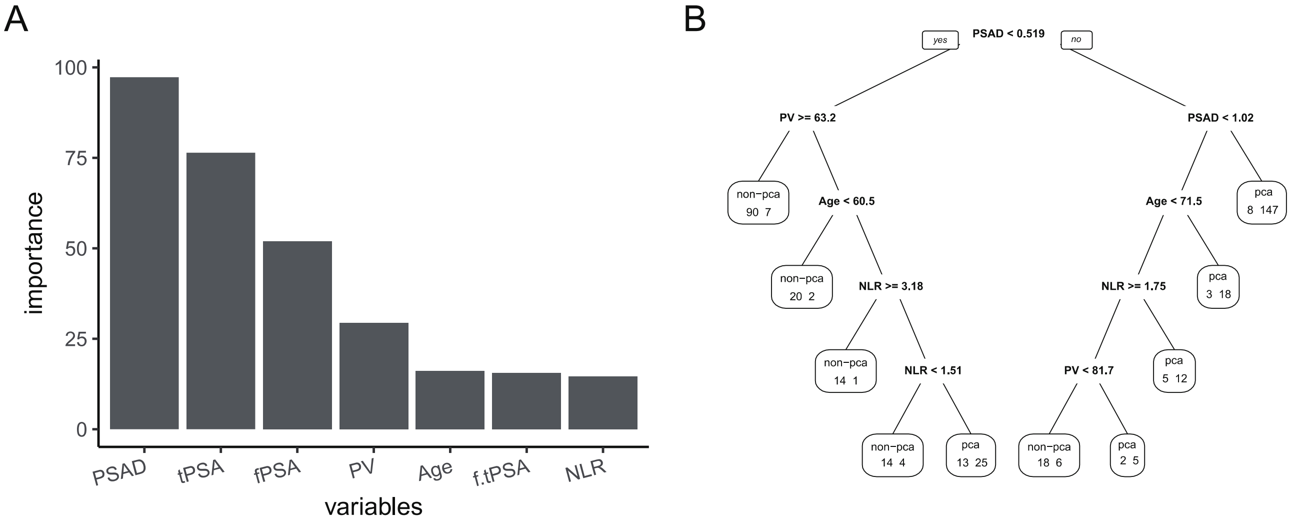

Supplementary Fig. 2 Analysis of the DT model. (A) The importance ordering of independent variables. The importance decreases from left to right. (B) Classic decision tree model. The optimal tree is the tree divided nine times (ten terminal nodes). Starting at the top of the tree, go left if the condition is true, go right otherwise, and the classification ends when the observation point reaches the end node. For prostate cancer ("pca" box), the number on the right in the box represents the correct diagnosis, and the number on the left represents the wrong diagnosis. For non-prostate cancer ("non-pca" box), the number on the left in the box represents the correct diagnosis, and the number on the right represents the incorrect diagnosis.

A B

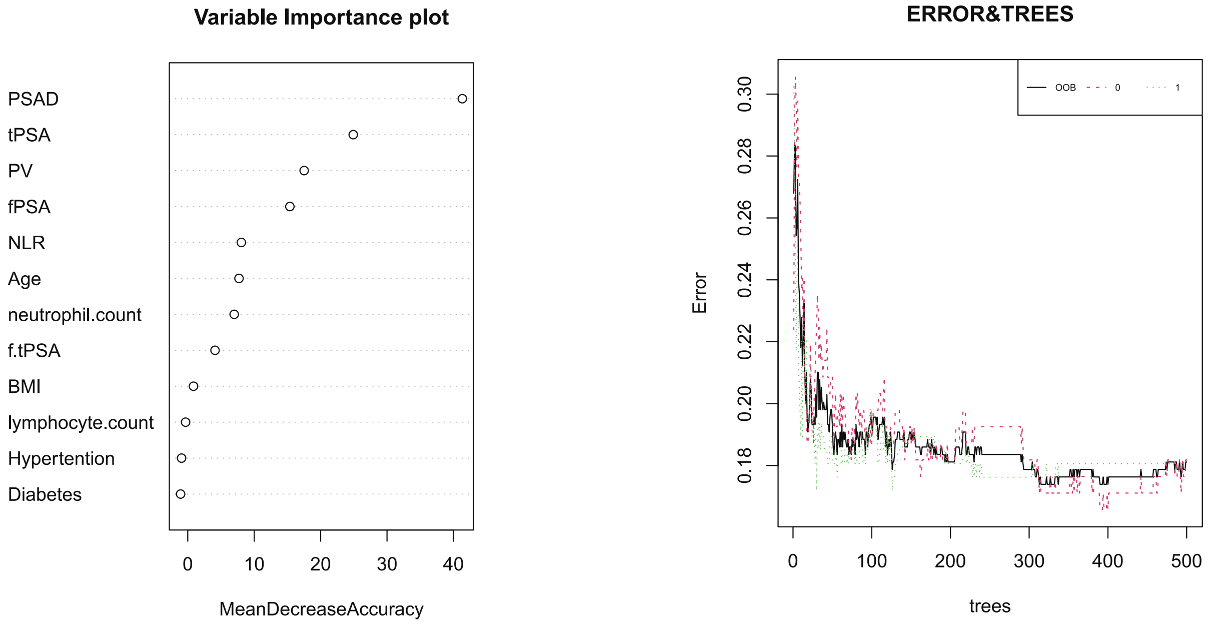

Supplementary Fig. 3 Analysis of the RF model. (A) Variable importance plot of the RF model. The importance increased with increasing value. (B) Error and trees plot. The plot demonstrates the relationship between RF model error and the number of decision trees. The red line represents patients without prostate cancer, the green line represents patients with prostate cancer, and the black line represents all patients.
